# Quarternary Structure and Enzymological Properties of the Different Hormone-Sensitive Lipase (HSL) Isoforms

**DOI:** 10.1371/journal.pone.0011193

**Published:** 2010-06-17

**Authors:** Christian Krintel, Cecilia Klint, Håkan Lindvall, Matthias Mörgelin, Cecilia Holm

**Affiliations:** 1 Department of Experimental Medical Science, Lund University, Lund, Sweden; 2 Division of Diabetes, Metabolism and Endocrinology, Department of Molecular Biophysics, Lund University, Lund, Sweden; 3 Division of Infection Medicine, Department of Clinical Sciences, Lund University, Lund, Sweden; University Paris Diderot-Paris 7, France

## Abstract

**Background:**

Hormone-sensitive lipase (HSL) is a key enzyme in the mobilization of energy in the form of fatty acids from intracellular stores of neutral lipids. The enzyme has been shown to exist in different isoforms with different molecular masses (84 kDa, 89 kDa and 117 kDa) expressed in a tissue-dependent manner, where the predominant 84 kDa form in adipocytes is the most extensively studied.

**Methodology/Principal Findings:**

In this study we employed negative stain electron microscopy (EM) to analyze the quarternary structure of the different HSL isoforms. The results show that all three isoforms adopt a head-to-head homodimeric organization, where each monomer contains two structural domains. We also used enzymatic assays to show that despite the variation in the size of the N-terminal domain all three isoforms exhibit similar enzymological properties with regard to psychrotolerance and protein kinase A (PKA)-mediated phosphorylation and activation.

**Conclusions/Significance:**

We present the first data on the quaternary structure and domain organization of the three HSL isoforms. We conclude that despite large differences in the size of the N-terminal, non-catalytic domain all three HSL isoforms exhibit the same three-dimensional architecture. Furthermore, the three HSL isoforms are very similar with regard to two unique enzymological characteristics of HSL, i.e., cold adaptation and PKA-mediated activation.

## Introduction

Hormone-sensitive lipase (HSL) is best known for its role in mobilizing fatty acids from triacylglycerol stores in adipocyte, although recent data suggest that it also plays an important role as a retinyl ester hydrolase [1]. In addition to the predominant, and most thoroughly investigated 84 kDa isoform found in adipocytes (HSL_adi_), HSL also exists in two other isoforms. In testis a considerably larger isoform of 117 kDa (HSL_tes_) is expressed [2], [3], and in insulin secreting β-cells an HSL isoform of 89 kDa (HSL_beta_) has been demonstrated [4]. HSL_tes_ appears to be exclusively expressed in testis [2], [3]. HSL_beta_, on the other hand, was cloned from insulin-producing β-cells [4], but is also expressed in several other cells, including adipocytes. In fact, most HSL-expressing cells appear to co-express HSL_adi_ and HSL_beta_ at different relative levels.

In the HSL gene nine exons, denoted 1–9, encode the sequences that are common to all three HSL isoforms. Upstream of these, exons B, A and T are located, each with its own promoter. These three upstream exons are mutually exclusive and joined to exons 1–9 by alternative splicing, forming transcripts for the functional HSL_adi_, HSL_beta_ and HSL_tes_ isoforms [3], [5], [6]. Whereas exon B is non-coding, exon A encodes an additional 43 N-terminal amino acids of HSL_beta_ with a high content of positively charged residues [5]. Exon T encodes an additional 300 amino acid sequence of HSL_tes_ with an unusually high content of proline and glutamine residues [3]. The three-dimensional structure has not been solved for any of the HSL isoforms, but several studies suggest a domain organization with two major structural domains, a 48 kDa C-terminal domain containing the active site in an α/β-hydrolase fold [7], [8], [9] and a 36 kDa N-terminal domain of HSL_adi_ that has been shown to complex with fatty acid binding protein [10], [11].

A unique feature among lipases is the regulation of HSL by reversible protein phosphorylation. This feature has been studied in extensively for HSL_adi_. The C-terminal domain comprises, in addition to the catalytic core, a 150 amino acid regulatory module including at least four serine phosphorylation sites namely Ser563, Ser565, Ser659 and Ser660 (HSL_adi_ numbering). *In vivo* Ser563, Ser659 and Ser660 are phosphorylated by PKA after stimulation of lipolysis. *In vitro* phosphorylation of Ser659 and Ser660 by PKA has been shown to increase the activity of the enzyme towards lipid substrates [7], [12], [13], [14], [15]. It has also been suggested that HSL dimerization is induced by phosphorylation [16]. Another unique feature of HSL is its high relative activity at low temperatures, a feature that has been suggested to be important in hibernating animals [17]. The structural basis of this cold adaptation property, which only has been studied for HSL_adi_, is not known.

The aim of this study was to characterize the different HSL isoforms with regard to quarternary structure and enzymological properties. Using negative stain EM we demonstrate that HSL predominantly exists as a homodimer, where each monomer of the dimer adopts a two-domain structure. This quarternary structure is preserved in all three isoforms. Furthermore, we show that the three isoforms are equally cold adapted and that *in vitro* phosphorylation and activation by PKA is not influenced by the increased size of the N-terminal domains in HSL_beta_ and HSL_tes_.

## Results

### Expression and purification of the three HSL isoforms

**Figure 1:**
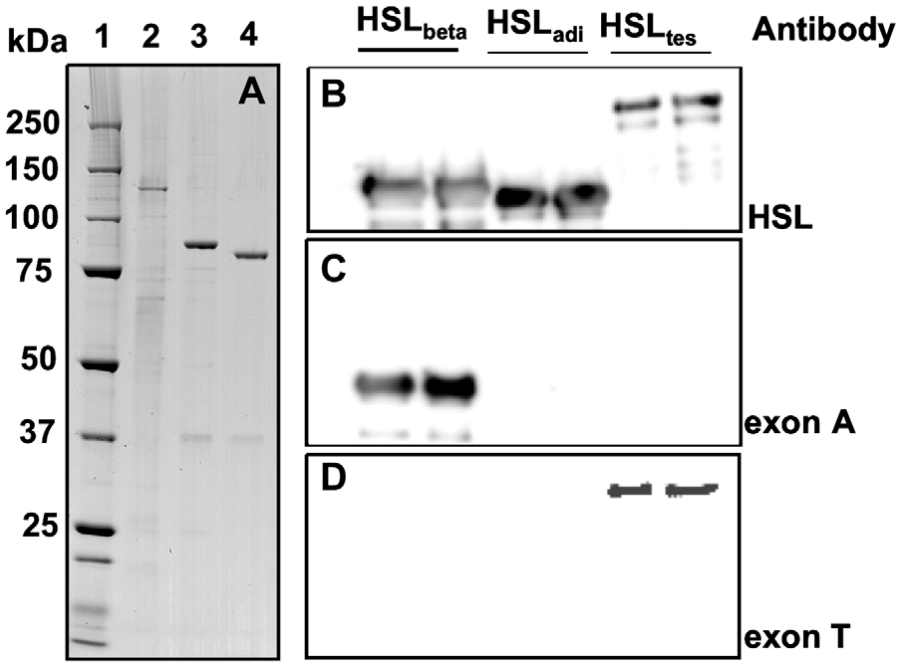
Purity and identity of recombinant HSL isoforms. Purified recombinant proteins were analysed by SDS-PAGE (A) and western blot (B–D). **(A)** Lane 1: MWM, lane 2: purified HSL_tes_, lane 3: purified HSL_beta_, lane 4: purified HSL_adi_. The identity of the respective purified isoforms was verified by western blot analysis using polyclonal antibodies generated against full-length HSL_adi_ **(B)**, polyclonal antibodies directed against amino acids encoded by exon A **(C)**, or polyclonal antibodies recognizing the amino acid sequence encoded by exon T **(D)**.

All three HSL isoforms were expressed as C-terminally His-tagged proteins in the baculovirus/insect cell system and purified by affinity chromatography. The purity of the protein preparations was approx. 95% for HSL_adi_ and HSL_beta_ and somewhat lower for HSL_tes_ as assessed by SDS-PAGE (**Figure 1A**). The identity of each isoform preparation was verified by western blot analysis, using polyclonal antibodies recognizing the sequence encoded by exons 1–9 of all HSL isoforms or antibodies specifically recognizing exon A-encoded sequences of HSL_beta_ or exon T-encoded sequences of HSL_tes_ (**Figure 1** **, B–D**). The specific activities obtained after purification was measured using pNPB as substrate. The obtained activities were 113 U/mg for HSL_adi_, 184 U/mg for HSL_beta_ and 26.5 U/mg HSL_tes_. These activites are lower than those reported for non-tagged rat HSL_adi_ but in accordance with results for C-terminally His-tagged human and rat HSL_adi_ [14], [15], [18].

### Transmission electron microscopy analysis

**Figure 2:**
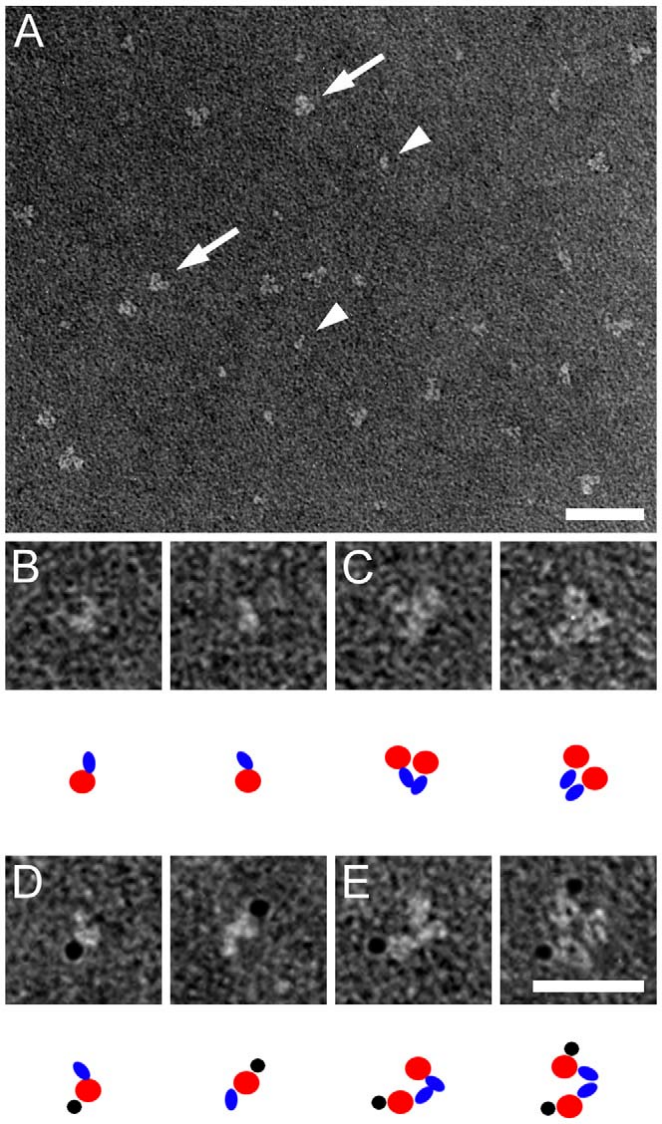
Negative stain electron microscopy analysis of HSL_adi_. Electron microscopy images depicting HSL_adi_ either native **(A–C)** or with Au-labelled anti-his Fab framents **(D–E)**. Partials **(B–E)** show either monomers **(B and D)** or dimers **(C and E)** in different orientations. As a guide to the TEM images, cartoons are included below each subpanel, showing the large domain in red, the small domain in blue and the gold-labelled Fab fragment in black. Scale bars are 50 nm **(A)** and 25 nm **(B–E)**. Au particles have a diameter of 3 nm.

**Figure 3:**
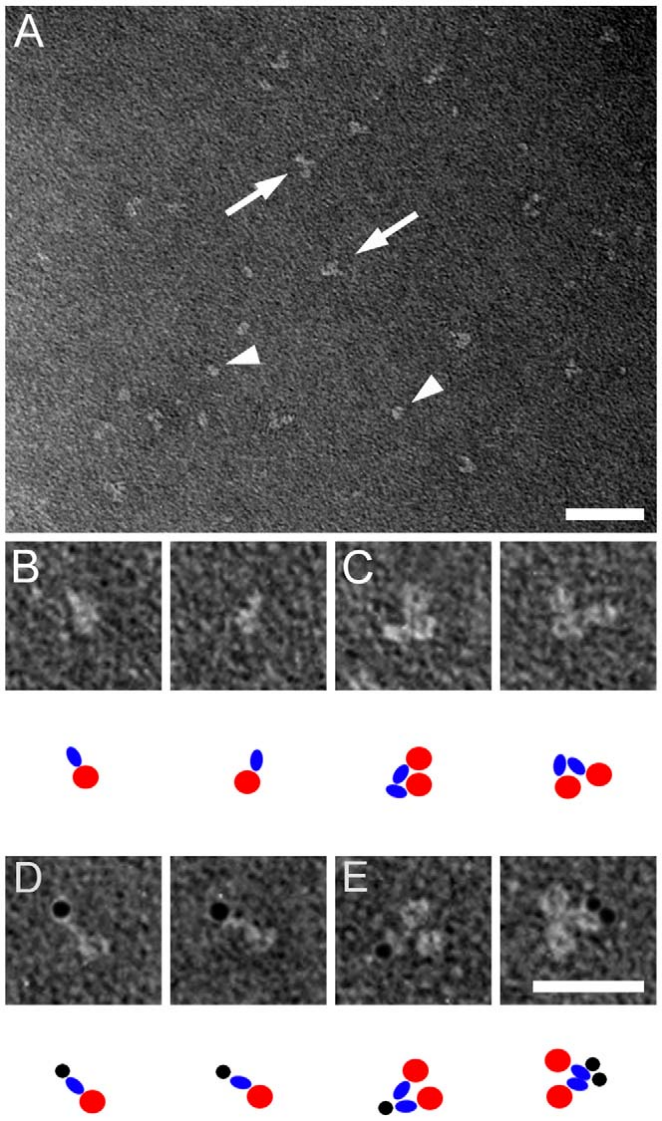
Negative stain electron microscopy analysis of HSL_beta_. Electron microscopy images depicting HSL_beta_ either native **(A–C)** or with Au-labelled anti-exon A Fab fragments **(D–E)**. Partials **(B–E)** show either monomers **(B and D)** or dimers **(C and E)** in different orientations. As a guide to the TEM images, cartoons are included below each subpanel, showing the large domain in red, the small domain in blue and the gold-labelled Fab fragment in black. Scale bars are 50 nm **(A)** and 25 nm **(B–E)**. Au particles have a diameter of 3 nm.

**Figure 4:**
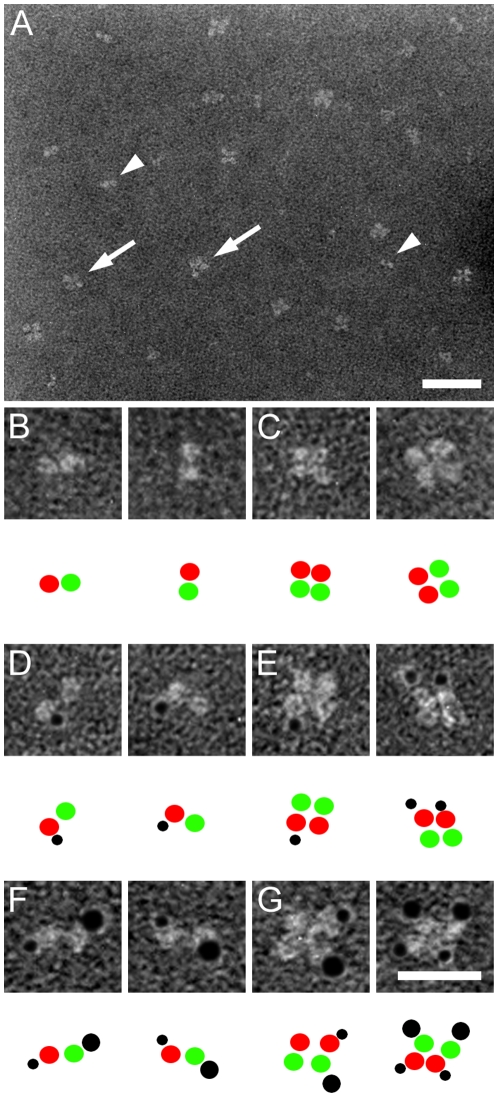
Negative stain electron microscopy analysis of HSL_tes_. Electron microscopy images depicting HSL_tes_ either native **(A–C)** or with Au-labelled anti-His-Fab fragments only **(D and E)** or in combination with Au-labelled anti-exon T- Fab fragments **(F and G)**. Partials **(B–G)** show either monomers **(B, D and F)** or dimers **(C, E and G)** in different orientations. As a guide to the TEM images, cartoons are included below each subpanel, showing the domain corresponding to the large domain in Figs. 2 and 3in red, the other domain in green and the gold-labelled antibodies in black. Scale bars are 50 nm **(A)** and 25 nm **(B–G)**. Au particles have a diameter of 3 nm (Anti-His_6_) or 5 nm (Anti-exon T).

Negative stain EM analysis revealed that all three HSL isoforms were found predominantly as homodimers (**Figure 2A** **,** **Figure 3A** **and** **Figure 4A**). Each monomer of these homodimers consisted in each case of two structural domains. For the HSL_beta_ and HSL_adi_ isoforms the two domains are different in size, whereas for HSL_tes_ the size of the two domains is similar (**Figure 2, B–C** **,Figure 3, B–C,** **Figure 4, B–C and** **Table 1**). The size distribution of the two domains in each isoform corresponds to their respective molecular weight. The largest domain of HSL_beta_ and HSL_adi_ co-localizes with gold-labelled anti-his Fab-fragments for HSL_adi_ (**Figure 2, D–E**) and the smaller domain with gold-labelled exon A Fab-fragments for HSL_beta_ (**Figure 3, D–E**) indicating that the larger domain of HSL_beta_ and HSL_adi_ is the catalytic domain.

**Table 1:** Quantification of TEM images (Figs. 2–4).

HSL isoform	Large domain	Small domain	Head-to-head arrangement (by immunogold)
HSL_adi_	6+/−1 nm	4+/−1 nm	89.5%
HSL_beta_	6+/−1 nm	4+/−1 nm	92.7%
HSL_tes_	6+/−2 nm	6+/−2 nm	90.2%

For each HSL isoform 600 particles were analyzed with regard to size of the two structural domains in the monomer and for presence of a head-to-head arrangement of the dimer. Large and small domain refer to the situation in HSL_adi_ and HSL_beta_, where the labeling pattern shows that the larger domain corresponds to the C-terminal catalytic domain and the small domain to the N-terminal non-catalytic domain.

In addition to information on the domain structure organization, the EM images also provided information on the orientation of the subunits in the HSL dimer. EM images shown in **Figure 2** and **Figure 3**, indicated that the monomers of the dimer of the smallest isoform (HSL_adi_) are oriented in a head-to-head fashion. To further verify this we used the larger HSL_tes_ isoform, which enabled us to distinguish two different sized Au labels without loosing the ability to observe the protein. Thus, HSL_tes_ was visualized in complex with 3 nm Au particle labelled anti-his antibodies and 5 nm Au particle labelled antibodies against exon T-derived sequences (**Figure 4** **, F–G**) to verify that the monomers indeed are oriented head-to-head in the dimeric assembly. Quantification of the TEM images in Figs. 2–4 showed that for each isoform a head-to-head arrangement of the two monomers in the dimer was found in 90% of the 600 particles analyzed (Table 1).

### Cold adaptation of HSL isoforms

**Figure 5:**
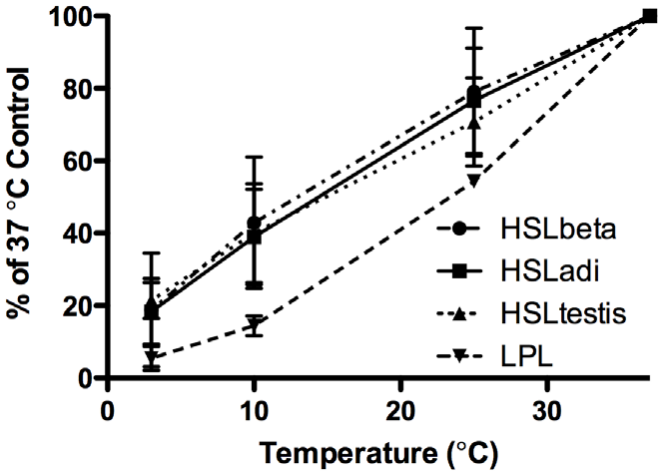
Cold adaptation of HSL isoforms. Esterase activity towards the *p*-nitrophenolbutyrate (pNPB) substrate of the three HSL isoforms and bovine lipoprotein lipase (LPL) at different assay temperatures. Results shown are mean ± SEM of seven or eight experiments (HSL isoforms) or two experiments (LPL) in triplicate.

Earlier reports have shown that HSL_adi_ has a remarkably conserved enzymatic activity at low temperatures. To test whether the size of the N-terminal domain had any influence on this enzymological property the enzymatic activity of all three HSL isoforms was assayed at 37°C, 25°C, 10°C and 4°C towards the pNPB substrate. All three isoforms were shown to have a similar preservation of enzymatic activity with decreasing temperature (**Figure 5**). The relative activity of lipoprotein lipase was lower than that of HSL at all temperatures assayed below 37°C.

### PKA phosphorylation of HSL isoforms

**Figure 6:**
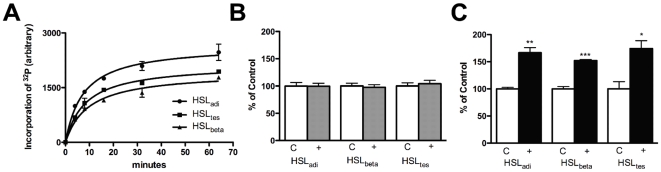
Phosphorylation and dephosphorylation of HSL isoforms purified from Sf9 cells. HSL_adi_, HSL_beta_ and HSL_tes_ were phosphorylated by PKA in the presence of ^32^P-labeled ATP. Reactions were subjected to SDS-PAGE and the amount of incorporated phosphate was determined by autoradiography and scintillation counting of gel bands followed by calculation from standards made from the original reactions **(A)**. HSL_adi_, HSL_beta_ and HSL_tes_ were dephosphorylated by calf intestinal phosphatase and assayed using triolein as substrate. Data represents mean ± SEM of six assays **(B)**. HSL_adi_, HSL_beta_ and HSL_tes_ were phosphorylated by PKA and assayed using triolein as substrate. Data represents mean ± SEM of triplicate assays. *P<0.05; *** P<0.0005, unpaired t-test **(C)**.

In order to investigate the extent of background phosphorylation of HSL purified from Sf9 insect cells, aliquots of preparations of purified HSL isoforms were incubated with calf intestinal phosphatase before assaying triglyceride lipase activity. In no experiment was any effect of this incubation detected on HSL activity (**Figure 6B**). This is in agreement with results from corresponding experiments performed on human HSL purified from Sf9 cells [14].

The extent of *in vitro* phosphorylation by PKA of the different HSL isoforms was investigated by performing phosphorylation experiments in the presence of ^32^P-labeled ATP. The stoichiometry of the phosphorylation was 0.22, 0.16 and 0.17 mol phosphate/mol HSL (**Figure 6A**). Phosphorylation by PKA increased the activity against triolein (TO) by 67%, 52% and 74% for the HSL_adi_, HSL_beta_ and HSL_tes_ isoforms, respectively (**Figure 6C**). Both phosphorylation and activation levels are in accordance with previously published levels for his-tagged rat and human HSL_adi_ [14], [15]. Since the degree of activation in absolute terms varied from experiment to experiment, representative results of several experiments (n = 3) are shown in **Figure 6C**. In all experiments the activity of the three isoforms increased in a similar manner after incubation with PKA.

## Discussion

The three different HSL isoforms were expressed successfully in Sf9 insect cells and purified (**Figure 1**). Using pNPB as substrate the specific activities of the obtained protein preparations differed when comparing the three isoforms, with a remarkably lower activity of HSL_tes_ compared to both HSL_adi_ and HSL_beta_. The lower activity of the HSL_tes_ preparations is most likely due to the lower purity obtained for this isoform. Attempts to increase the purity of this isoform with retained activity were in our hands unsuccessful. HSL_adi_ and HSL_beta_, on the other hand, were purified to homogeneity with a higher activity of HSL_beta_ than HSL_adi_. The reason for the higher specific activity of HSL_beta_ towards pNPB than that of HSL_adi_ is not clear, but the difference was consistent through all preparations.

Mutagenesis studies of the C-terminal, catalytic domain of HSL_adi_ has failed to identify the structural basis for the cold adaptation of HSL and provide an explanation for the more pronounced cold adaptation of rat HSL_adi_ compared to human HSL_adi_ [17], raising the possibility that the N-terminal domain impact the psychrotolerant properties of HSL. We therefore compared the activities of the three isoforms at decreasing temperatures. However, our results clearly demonstrate that all three HSL isoforms exhibit the same degree of psychrotolerance, suggesting that this feature of HSL indeed is determined by the catalytic domain. Atomic structures of both rat and human HSL are needed to confirm this and to identify the structural features involved in cold adaptation.

It is well established that phosphorylation by PKA *in vitro* increases the activity of HSL_adi_ against TO [7], [12], [14], [15]. Recent studies have shown that the N-terminal domain of HSL docks with lipid binding proteins in a phosphorylation-dependent manner, raising the possibility that the size of the N-terminal part of HSL could modulate HSL activation. Using radiolabelled ATP it was demonstrated in this study that there were very small, if any, differences in the degree of *in vitro* phosphorylation of the three HSL isoforms, indicating that the size of N-terminal domain does not influence the interaction between PKA and HSL. Accordingly, the PKA-mediated *in vitro* activation was also very similar for the three isoforms. Thus, it is reasonable to assume that PKA takes part in the regulation of HSL activity not only in adipocytes and skeletal muscle, but also in both beta-cells and testis. This in line with previously published studies illustrating the importance of PKA in regulation of pancreatic beta-cell function and spermatogenesis [19], [20], [21].

The quarternary structure of HSL is well conserved in all isoforms (**Figure 2A** **,** **Figure 3A** **and** **Figure 4A**). Conservation of a homodimeric structure is consistent with the concept that dimerization is important for HSL function and the smallest functional unit of HSL could thus be the homodimer. In addition, it was shown, with the aid of different sized gold labels on two different Fab fragments, that the monomers in the homodimer are arranged in a head-to-head fashion (**Figure 4, F–G**). This is in contrast to what has previously been shown for two other mammalian lipases, lipoprotein lipase and endothelial lipase, where the two monomers in the dimer are oriented in a head-to-tail fashion, where a lipid-binding, non-catalytic C-terminal domain, presents the lipid substrate to the catalytic domain of the other monomer of the dimer [22], [23]. The observation that the degree of dimerization of the HSL preparations was very high, despite that little background phosphorylation was observed, argues against that dimerization of HSL is brought about by phosphorylation as previously suggested [16].

Even though we did not find any differences between the different HSL isoforms with regard to the structural and enzymological properties studied here, it is possible, or even likely, that functional differences exist. One possibility is that the N-terminal domain is involved in protein-protein interactions and/or in localizing the enzyme to specific subcellular compartments. The N-terminal domain of HSL_adi_ has in fact been shown to interact with FABP. Depending on the hormonal status of the adipocyte, HSL_adi_ is located either in the cytsol (hormonally queiscent cells) or at the lipid droplet (β-adrenergic stimulation) [24], whereas HSL_beta_ has been found to be located in close association to insulin granules of β-cells [5]. The subcellular localization of HSL_tes_ is not known, but based on studies performed in HSL null mice it can be speculated that HSL_tes_ is indirectly associated with the membrane in haploid germ cells, where it controls cholesterol deposition and thus membrane fluidity [25].

In conclusion, we here present the first data of the quarternary structure of HSL. All three known HSL isoforms exhibit a homodimeric structure with a head-to-head orientation of the two monomers, and with two major structural domains in each monomer. We also demonstrate that despite large variations in the size of the N-terminal domain do the three HSL isoforms exhibit the same degree of psychrotolerance and the same degree of PKA-mediated phosphorylation and activation *in vitro*. These results suggest that the N-terminal domain of HSL may only play a role in modulating the in vivo activity of HSL, for instance through mediation of protein-protein interactions.

## Materials and Methods

### Generation of recombinant baculovirus transfer vectors

The 5′-sequences of HSL_tes_ and HSL_beta_ cDNA [3], [5] were obtained by PCR using the antisense primer 5′-ATA GAC TCC GTA AGC GCC CAG AAT-3′, the sense primers 5′-CAC AGG ATC CAG GAT GAA ACC TAG GAG ACC-3′ (BamHI) and 5′-GTG GAT CCA TGG AGC CGG CCG TGG AAT C-3′ (BamHI), respectively, and the corresponding cDNA as template. The obtained PCR products were digested using BamHI and each subcloned into the previously described pVL1393-HSL_adi_-His_8_ [15]

### Insect cell culture, transfection and expression of HSL isoforms

Sf9 insect cells (BD Pharmingen) were maintained in suspension culture at 27°C and 117 rpm in Sf-900 II SFM medium (Gibco-BRL) supplemented with 4% foetal calf serum (Gibco-BRL) and 100 U/ml penicillin/streptomycin (Gibco-BRL). Transfection of Sf9 cells was performed using the BaculoGold™ Transfection Kit (BD Pharmingen) according to the manufacturer's instructions. Plaque purification was performed and high titre virus stocks generated using standard procedures. Protein expression was performed according to [26].

### Purification procedures

Cells expressing either HSL_beta_ or HSL_adi_ were harvested by centrifugation (1200 g, 10 min), and resuspended in lysis buffer (40 mm Tris/HCl, pH 7.9, 1 mM β-mercaptoethanol, 1% C13E12, 10% glycerol, 20 mg/l leupeptin, 10 mg/l antipain and 1.0 mg/l pepstatin). The cell suspension was gently sonicated and centrifuged for 45 min at 4°C and 50 000 g.

Following addition of imidazole and NaCl to final concentrations of 5 mM and 0.3 M, respectively, the cell supernatant was mixed with Ni-NTA agarose (Qiagen) pre-equlillibrated in buffer A (20 mM Tris/HCl, pH 7.9, 0.3 M NaCl, 5 mM imidazole, 10% glycerol, 0.1% C_13_E_12_, 20 µg/l leupeptin, 10 µg/l antipain and 1.0 µg/l pepstatin). The Ni-NTA agarose and the applied supernatant were gently agitated for 60 min at 10°C followed by low-speed centrifugation and removal of the supernatant. The Ni-NTA slurry was washed twice in 10 volumes of buffer A containing 60 mM imidazole. Protein was subsequently eluted in buffer A containing 250 mM imidazole.

For purification of HSL_tes_ an alternate procedure was used. Instead of Ni-NTA agarose the cell supernatant was mixed with a TALON Resin (BD Biosciences), equilibrated in buffer T (0.25 M sucrose, 5 mM Tris/HCl, pH 7.9, 0.3 M NaCl, 5 mM imidazole, 1% C_13_E_12_, 1 mM β-ME, 20 µg/l leupeptin, 10 µg/l antipain, 1.0 µg/l pepstatin). After gentle agitation for 60 min at 10°C the resin was washed in 10 volumes of buffer T and 10 volumes of buffer T containing 7.5 mM imidazole. Protein was subsequently eluted in buffer T containing 100 mM imidazole.

Following purification the respective elution buffers were immediately exchanged for 10 mM Tris/HCl, pH 7.3, 0.14 M NaCl, 10% glycerol, 0.2% C_13_E_12_, 1 mM β-ME using a PD-10 desalting column (Amersham Biosciences). Protein concentration in preparations was determined using the 2-D Quant Kit (Amersham Biosciences).

### Antibodies for western blot

As primary antibody for all HSL isoforms a polyclonal rabbit anti-HSL antibody raised against the amino acid sequence encoded by exons 1–9 of rat HSL [8] was used. HSL_beta_ was selectively detected using a polyclonal rabbit antibody raised against the exon A-encoded sequence of HSL_beta_ [5], and for HSL_tes_ a polyclonal antibody against exon T-derived sequences was used [27].

### Transmission electron microscopy (TEM) and immuno-EM of HSL isoforms

The domain structure organization of the different HSL isoforms was analyzed by negative staining and transmission electron microscopy as described previously [28]. Fab fragments of antibodies against the His-tag (Qiagen) and the exon A-encoded sequence, respectively, were conjugated with 3 nm colloidal Au. Fab fragments of antibodies against the exon T-encoded sequence were conjugated with 5 nm colloidal Au [29]. HSL aliquots were mixed with the Au conjugates and incubated for 1 h at 4°C. Five microliter aliquots were adsorbed onto carbon-coated grids for 1 min, washed with two drops of water, and stained on two drops of 0.75% uranyl formate. The grids were rendered hydrophilic by glow discharge at low pressure in air. Specimens were observed in a JEOL JEM 1230 electron microscope operated at 80 kV accelerating voltage, and images were recorded with a Gatan Multiscan 791 CCD camera.

### HSL activity assays

HSL lipase activity was measured against phospholipid-stabilized emulsions of tri[^3^H]oleoylglycerol (TO) as previously described [8], [15], [30], [31]. Esterase activity of the HSL isoforms was measured using para-nitrophenyl (pNPB) butyrate as substrate (Sigma). For the temperature dependence experiments, assays were performed according to [32]. Assay conditions for purified bovine lipoprotein lipase [33] were adjusted according to [34].

### PKA phosphorylation of HSL isoforms

Four to six µg of the respective HSL isoform was phosphorylated essentially as described [15] with the exception that the buffer used in reactions was 50 mM Tris pH 8, 300 mM NaCl, 10% glycerol, 0.2% C_13_E_12_, 1 mM DTT, 0.2 mM ATP, 0.5 µCi/µl [−^32^P] ATP and 10 mM MgCl_2_, 0.25 U/µl PKA (New England Biolabs) supplemented with a cocktail of protease inhibitors (Roche Complete). Radiolabeled ATP was omitted in reactions used for activity assays. Dephosphorylation was performed as described in [14] with the exception that the reaction buffer was 50 mM Tris pH 8, 300 mM NaCl, 10% glycerol, 0.2% C_13_E_12_, 1 mM DTT, 10 mM MgCl_2_, 0.2 U/µl calf intestinal phosphatase (CIAP, Fermentas) supplemented with a cocktail of protease inhibitors (Roche Complete). Samples used for activity assays were assayed after 1 h of incubation with PKA or CIAP.
